# Splenic Artery Aneurysm in Gaucher Disease: A Hybrid Study Combining Case Report, Scoping Review, and Clinical Survey

**DOI:** 10.1002/jmd2.70044

**Published:** 2025-09-25

**Authors:** Paolo Manzi, Anita Vergatti, Veronica Abate, Nadia Altavilla, Michelina Sibilio, Pietro Venetucci, Paolo Tirelli, Domenico Rendina, Antonio Barbato

**Affiliations:** ^1^ Department of Clinical Medicine and Surgery University of Naples “Federico II” Naples Italy; ^2^ Metabolic Diseases Unit Santobono‐Pausilipon Children's Hospital Naples Italy; ^3^ Advanced Biomedical Sciences Department University Federico II of Naples Naples Italy; ^4^ Department of Internal Medicine “Ospedale del Mare” Hospital Naples Italy

**Keywords:** enzyme replacement therapy, Gaucher disease, lysosomal storage disease, splenic artery aneurysm, splenomegaly, thrombocytopenia

## Abstract

Gaucher disease (GD) is a rare lysosomal storage disorder caused by pathogenic variants in the *GBA* gene. Splenic artery aneurysm (SAA) is an uncommon and underrecognized complication of GD, particularly in the absence of traditional risk factors. SAA carries a high risk of rupture and significant mortality. This hybrid study investigates the clinical features and impact of SAA in GD through a three‐part approach: a case report, a scoping review with individual patient data (IPD) analysis, and a clinical survey conducted across two GD centers in Naples, Italy. We report the case of a 46‐year‐old woman with GD type 1, in whom a 37 × 32 mm calcified SAA was incidentally found. Despite the high risk of rupture, the patient opted for regular radiological monitoring. Over a 16‐year follow‐up period, the aneurysm remained stable. In the literature, we identified 11 GD subjects (four [36.4%] males and seven [63.6%] females) with SAA, including our case report. Most had not received enzyme replacement therapy (ERT) or were non‐compliant before the SAA diagnosis. Only two patients reported abdominal pain, and one died due to intraoperative hemorrhage. In our clinical survey, the prevalence of SAA in GD patients was 2.1%, twice that reported in individuals without GD. Although SAA is a rare and often underdiagnosed complication in GD, its potential lethality warrants heightened clinical awareness. We recommend incorporating magnetic resonance imaging for routine evaluation of the spleen and splenic artery in GD patients. This strategy, in conjunction with timely ERT, may be life‐saving.

**Summary**

- Splenic artery aneurysm (SAA) is a rare but clinically relevant complication of Gaucher disease (GD), likely related to the disease pathogenesis (splenomegaly, Gaucher cell infiltration, and chronic inflammation)

- Lack of or poor adherence to enzyme replacement therapy (ERT) or substrate reduction therapy (SRT) appears to increase the risk of SAA, while treatment may have a protective role

- Routine follow‐up of GD patients should include specific assessment of the splenic artery to improve early detection and reduce rupture‐related mortality.

## 1 Introduction

Gaucher disease (GD) is a rare, autosomal recessive lysosomal storage disease (LSD) caused by pathogenic variants in the glucosylceramidase beta 1 (*GBA*) gene, located on chromosome 1q21. GD is characterized by a deficiency of the lysosomal enzyme acid β‐glucosidase, leading to the accumulation of glucocerebroside within lysosomes of macrophages. The systemic accumulation of these glycolipid‐lipid engorged cells, known as Gaucher cells, typically results in splenomegaly, anemia, thrombocytopenia, hepatomegaly, and a complex array of skeletal manifestations that are often disabling. These include bone deformity, osteopenia, osteosclerosis, osteonecrosis, fractures, and bone marrow infiltration [1, 2].

Although the clinical phenotype of GD spans a continuous spectrum from mild to severe, three main clinical forms have been identified. Type 1, accounting for approximately 90% of cases in Europe and the USA, is characterized by visceral involvement, without central nervous system impairment. In contrast, types 2 and 3 are associated with neurological manifestations, severe and rapidly progressive in type 2, and more variable with later onset in type 3 [3].

GD was the first genetic condition for which intravenous enzyme replacement therapy (ERT) was developed. More recently, orally administered substrate reduction therapies (SRT), which inhibit glucosylceramide biosynthesis, have also become available [3].

A rare and poorly described complication associated with GD is splenic artery aneurysm (SAA), in the absence of other risk factors. SAA can lead to vessel rupture, carrying a high risk of mortality [4]. SAA is the most commonly reported type of visceral artery aneurysm, accounting for 60%–70% of diagnosed cases. Atherosclerosis, portal hypertension, liver transplantation, pregnancy, and connective tissue disorders are the main modifiable risk factors, whereas advanced age and female sex represent non‐modifiable ones. SAA is often asymptomatic; however, when symptoms do occur, they typically include vague epigastric or left upper quadrant abdominal pain. In some cases, gastrointestinal or pancreatic hemorrhage may be the only clinical manifestation. Although rare, rupture is a potentially life‐threatening complication, presenting with sudden abdominal pain, hypovolemic shock, and, in severe cases, death. The risk of rupture is significantly increased during pregnancy, particularly in multiparous women and in patients with portal hypertension [5].

The aim of our hybrid study is to describe the clinical characteristics and prognosis of this condition by presenting (1) a new case of SAA in a GD patient, (2) a scoping review with individual patient data analysis (IPD) to define clinical features, treatment approaches, and outcomes, and (3) a clinical survey conducted across two GD centers in the Campania region.

## 2 Materials and Methods

### 2.1 Case Report

The patient voluntarily signed informed consent to the publication of the case report.

### 2.2 Scoping Review With IPD Analysis

#### 2.2.1 Data Sources and Search Strategy

The review was planned, conducted, and reported according to the Preferred Reporting Items for Systematic Reviews and Meta‐Analyses (PRISMA) statement [6] (Figure S1). A systematic search was performed in Medline, Google Scholar, Google Books, and Cochrane Library (last conducted search January 20, 2025) using the following terms: “splenic artery aneurysm,” “Gaucher disease.” No language restrictions were applied. The reference lists of all identified articles were searched for additional relevant publications.

#### 2.2.2 Study Selection

Eligible studies included case reports and case series. All articles were obtained in full text, and the references were analyzed to exclude duplicate data.

#### 2.2.3 Data Extraction

A standardized, pre‐piloted form was used to extract relevant clinical data from the included studies by two independent authors (AV and VA). The extracted data included: (a) first author's last name, (b) publication year, (c) gender, (d) age, (e) age at the diagnosis of splenic artery aneurysm, (f) genotype, (g) GD type 1 therapy, (h) death, (i) age at death, (j) cause of death, (k) follow‐up, and (l) treatment. All disagreements related to the extracted data were resolved through a discussion with another author (DR). When available, missing data were obtained upon request from study authors via mail. The critical appraisal of case reports and case series included in the systematic review was conducted according to the Joanna Briggs Institute checklist for case series (Table S1) and for case reports (Table S2) [7].

### 2.3 Clinical Survey

From November 1st, 2024 to February 28th, 2025, all patients referred to the Department of Clinical Medicine and Surgery of the University of Naples and “Ospedale del Mare” (Naples, Italy) for GD were enrolled in our clinical survey. All GD patients underwent the evaluation of the spleen and splenic artery by abdominal ultrasound and Doppler assessment. SAA was defined according to criteria proposed by the American Society for Vascular Surgery [8]. A standardized pre‐piloted form was used to extract relevant data from enrolled patients' medical records. The extracted data included: (c) gender, (d) age, (e) age at the diagnosis of GD, (f) genotype, (g) GD type 1 therapy, and (h) comorbidities. Two authors (PM and AV) extracted data independently, and any discrepancies were resolved through discussion with author (AB). A post hoc sample size analysis indicated that the enrolled population was representative of all patients with GD type 1 living in Southern Italy, based on the estimated GD type 1 prevalence in Italy (0.89 per 100 000 inhabitants) reported by Carubbi et al. [9] and the total population of Southern Italy [10].

### 2.4 Statistical Analysis

All statistical analyses were performed using the IBM SPSS (Statistical Package for Social Science), version 27 (IBM, Armonk, NY, USA). Data from study publications were extracted and included in a single database. These data were then reanalyzed and combined. The *χ* ^2^ or Fisher exact test was used to evaluate differences between categorical variables or proportions. The Kolmogorov–Smirnov test was used to assess the data distribution. Data are expressed as mean ± standard deviation for continuous variables with a normal distribution, as median (25th–75th percentile) for continuous variables with not‐normal distribution, and as absolute numbers (percentages) for categorical variables [11]. All statistical tests were two‐tailed. A *p* value < 0.05 was considered statistically significant.

## 3 Results

### 3.1 Case Report

A 60‐year‐old woman with GD from Southern Italy, born to non‐consanguineous parents, had a medical history notable for chronic anemia and hypermenorrhea since adolescence, with a hospitalization at age 16 due to these conditions. At age 26, she was diagnosed with GD type 1 during an admission for asthenia, muscle pain, splenomegaly, and weight loss. Blood tests revealed severe anemia (hemoglobin: 58 g/L) and thrombocytopenia (platelet count: 35 000/mm^3^). A bone marrow aspirate was performed, showing foamy, vacuolated cells with eccentric nuclei consistent with Gaucher cells. The diagnosis was confirmed by markedly reduced acid β‐glucosidase activity in peripheral leukocytes and molecular analysis showing compound heterozygosity for *GBA* gene pathogenic variants: (c.1226A>G p.(*Asn409Ser*)) and (c.894C>A p.(Phe298Leu)).

**FIGURE 1:**
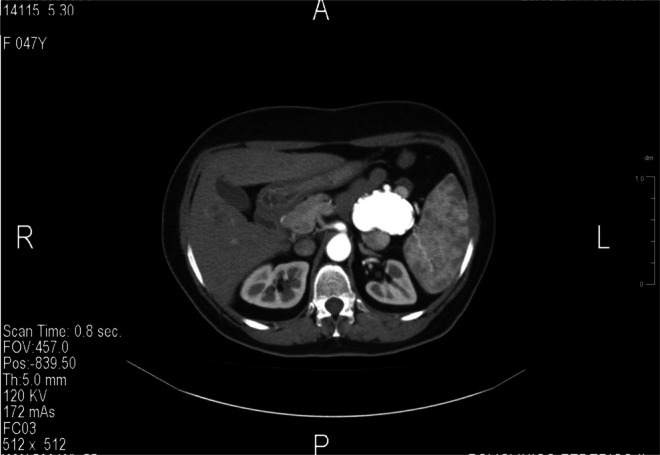
Computed tomography scan of the third lateral splenic artery aneurysm.

**FIGURE 2:**
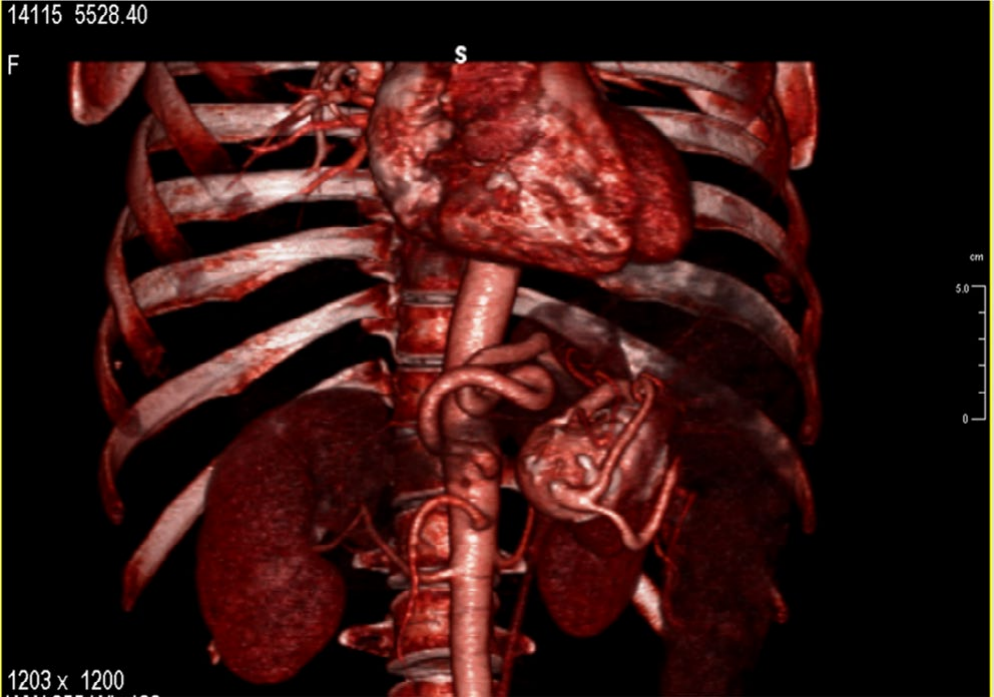
Three‐dimensional reconstruction of abdominal computed tomography scan.

Until the age of 36, when ERT became available in Italy, the patient was treated with iron and vitamin supplementation. She then started ERT with Imiglucerase at 60 U/kg, which was gradually reduced to 30 U/kg over time, achieving a good response in hematological and visceral parameters. At age 46, she began follow‐up at our Department of Clinical Medicine and Surgery, University of Naples. In accordance with GD monitoring recommendations [4], she underwent abdominal CT (MRI being contraindicated due to claustrophobia), which revealed a thin‐walled, calcified SAA measuring 37 × 32 mm (Figures 1 and 2). The patient was informed of the potential risk of aneurysm rupture [12], but she declined surgical or endovascular intervention and opted for close imaging follow‐up. The aneurysm remained stable over the years.

Regarding laboratory tests, platelet count and hemoglobin levels normalized, and splenomegaly progressively reduced, from 174 mm longitudinal diameter (volume: 434 cc) at age 50–124 mm (volume: 220 cc). However, an elevation in biomarkers such as chitotriosidase (115 nmol 4 MU/mL/h; normal range 3.2–97.2) and Lyso‐GL1 (46.4 ng/mL; normal range 0–14) persisted.

### 3.2 Scoping Review With IPD Analysis

As shown in Figure S1, we have included six studies. There were four case reports and two case series included in the analysis [13, 14, 15, 16, 17, 18]. A complete list of studies included and their quality evaluation is reported in Tables S1 and S2. Eleven subjects (male: female [M: F] = 4 [36.4%]: 7 [63.6%]; mean age 46.3 ± 10.7 years) affected by GD and SAA, including our case report, were described from 1942. No significant differences were found between males and females. Seven subjects (M: F = 3 [42.9%]: 4 [57.1%]) did not receive ERT before the diagnosis of SAA. Nine subjects (M: F = 4 [44.4%]: 5 [55.6%]) were affected by splenomegaly and eight (M: F = 4 [50.0%]: 4 [50.0%]) by thrombocytopenia. Two females (18.2%) showed symptoms, such as abdominal pain. Only one individual (9.1%) died due to an acute and catastrophic intraabdominal hemorrhage, and SAA was identified post‐mortem. She had not received ERT for about 10 years before SAA. The main treatments were represented by splenectomy (4; 36.4%) and embolization (1; 9.1%), whereas two patients (18.2%), including our case, chose close monitoring. The mean follow‐up was 5.8 ± 6.6 years. Extracted data regarding patients included in the Scoping review with IPD analysis are reported in Table S3.

### 3.3 Clinical Survey

We have collected data from 48 patients (M: F = 19 [39.6%]: 29 [60.4%]; mean age 50.2 ± 16.5 years) affected by GD. Forty‐five GD patients were treated with ERT (mean duration of treatment 22.24 ± 9.4 years) and three with SRT (mean duration of treatment 2.2 ± 0.5 years) and underwent clinical, laboratory, and instrumental follow‐up for GD. Only one patient developed an SAA. The prevalence of SAA was 2.1% (1/48).

## 4 Discussion

The results of our hybrid study show that, although rare, SAA may represent a potential complication of GD, and the occurrence appears to be more frequent in patients who did not receive or were non‐compliant with ERT or SRT prior to the diagnosis of SAA. Notably, the prevalence in our clinical survey is consistent with previously reported data (2%, 5/252) [13].

Since 1942, different cases of SAA have been described in subjects with GD, suggesting a possible predisposition to aneurysm formation [13, 14, 15, 16, 17, 18]. The prevalence of SAA in GD appears to be higher than in the general population (less than 1%), despite being frequently underdiagnosed [5]. Unlike patients with GD, who routinely undergo specialist evaluations to monitor disease progression, most individuals in the general population do not undergo instrumental assessment of the spleen. Diagnosis via abdominal ultrasonography remains challenging, due to the deep course and the tortuous shape of the splenic artery in GD [19]. Moreover, this condition remains asymptomatic until the aneurysm reaches giant or near‐giant dimensions [15]. Taken together, these observations suggest that GD can be considered as a possible and independent risk factor for SAA.

Other arterial aneurysms, including aortic, splenic, iliac, and intracranial, have also been reported in GD; pathophysiological mechanisms remain poorly understood. Several hypotheses have been proposed, including hepato‐splenomegaly with venous hypertension, infiltration of Gaucher cells, and chronic inflammation [13, 20, 21]. Hepato‐splenomegaly may increase vascular pressure and promote turbulent blood flow [13]. The infiltration of Gaucher cells may compromise the structure of the vascular wall, leading to its weakening [22]. Finally, chronic inflammation, caused by glucocerebrosidase accumulation, may increase cytokines and matrix metalloproteinases, contributing to vessel wall vulnerability [13].

As suggested by our results, ERT or SRT may protect against SAA formation and enlargement. Indeed, both ERT and SRT are effective to reduce liver and spleen volume, Gaucher cell infiltration, and chronic inflammation [23]. All these elements affect the pathogenesis of SAA, and treating GD by reducing splenomegaly, inflammation, and glucocerebroside accumulation is likely to have a positive impact on aneurysm‐related outcomes as well. However, there is no evidence that ERT is protective against aneurysm rupture in subjects with already diagnosed SAA.

SAA is typically asymptomatic and often underdiagnosed, with an elevated risk of mortality if not treated promptly (about 75% of symptomatic subjects). While open surgery remains the gold standard for SAA repair, endovascular approaches have demonstrated high success rates, and laparoscopic repair is a safe option for elective cases. Due to the risk of aneurysmal rupture, intervention is advised in case of SAA greater than 2 cm in diameter [8]. In severe cases (e.g., hostile abdomen, distal aneurysms near the splenic hilum, or in case of unfeasible hemostasis), splenectomy may be required [5]. However, as demonstrated in our case report, a conservative approach involving ERT or SRT combined with close imaging surveillance may be a safe alternative. Any non‐surgical approach should involve a shared decision‐making process between the patient and their clinical team. Factors such as the patient's ability to adhere to therapy, overall clinical condition, comorbidities, lifestyle, and specific risk factors for rupture must be taken into account.

Our hybrid study suggests the necessity of in‐depth assessment of the splenic artery in GD patients, given the elevated risk of SAA and the high mortality associated with its rupture. Current recommendations already suggest regular imaging follow‐up in GD patients, performed annually with abdominal ultrasound and magnetic resonance imaging, to assess spleen and liver volumes [24]. Based on our findings, we propose a more comprehensive evaluation that also includes the splenic artery, with the potential to reduce the risk of underdiagnosis. This is particularly relevant for GD patients with marked splenomegaly or signs of portal hypertension, as well as for pregnant women, in whom more detailed imaging may provide additional clinical value, especially when ultrasound findings are inconclusive. This screening strategy combined with ERT or SRT may prove to be life‐saving in GD patients.

Our study presents some limitations and strengths. A major limitation is the study design, which does not allow us to demonstrate a causal relationship between the lack of ERT and the occurrence or progression of SAA. Another limitation is the small sample size, which prevents us from performing subgroup analyses according to genotype, sex, or age. Furthermore, we are not able to provide a reliable estimate of the prevalence of SAA among patients with GD. Among the strengths of our study, we highlight the hybrid design, which combines a comprehensive review of the available literature with a clinical survey conducted on a representative sample of the entire GD population living in Southern Italy [10].

Ultimately, our study may serve as a reference point for future investigations, engaging leading centers involved in the care of GD. Of course, a multicenter study or a nationwide survey could provide more comprehensive data on the actual prevalence of SAA in this population.

## Author Contributions

**Paolo Manzi:** investigation, writing – original draft preparation. **Anita Vergatti:** conceptualization, writing – original draft preparation, writing – review and editing. **Veronica Abate:** resources. **Nadia Altavilla:** resources. **Michelina Sibilio:** resources. **Pietro Venetucci:** resources. **Paolo Tirelli:** visualization. **Domenico Rendina:** methodology, visualization. **Antonio Barbato:** conceptualization, supervision. All authors have read and agreed to the published version of the manuscript.

## Consent

The patient voluntarily signed informed consent.

## Conflicts of Interest

The authors declare no conflicts of interest.

## Supporting information


**Figure S1:** Flow chart of study selection (PRISMA).

**Table S1:** Quality assessment for case reports.

**Table S2:** Quality assessment for case series.

**Table S3:** Characteristics of subjects included in the Scoping review with IPD analysis.

## Data Availability

All data supporting the findings of this study are available from the corresponding author upon reasonable request.
