# Supplementary figures and images for: Splenic Artery Aneurysm in Gaucher Disease: A Hybrid Study Combining Case Report, Scoping Review, and Clinical Survey
Source: JIMD Rep. 2025 Sep 25;66(6):e70044. doi: 10.1002/jmd2.70044 (PMC12464341; doi:10.1002/jmd2.70044)

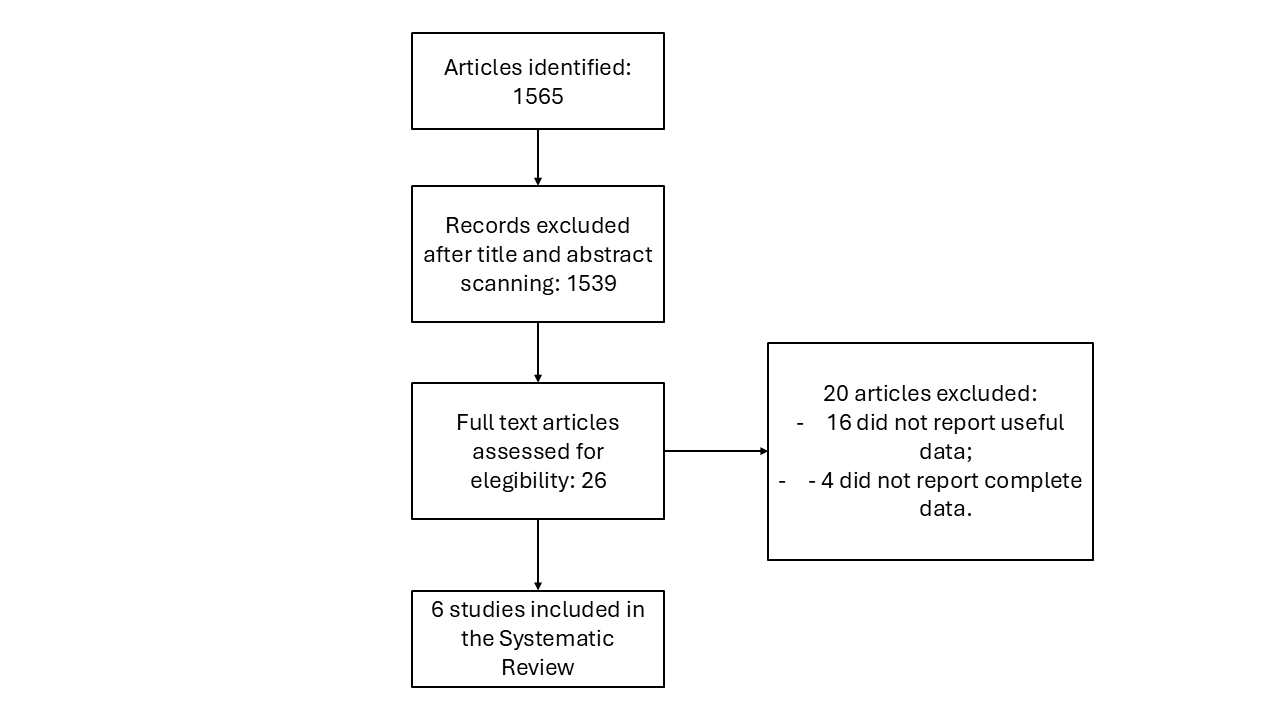

Supplement: Supplementary file 1 — Figure S1: Flow chart of study selection (PRISMA). [file JMD2-66-e70044-s004.tif]
