# Supplementary material for: Splenic Artery Aneurysm in Gaucher Disease: A Hybrid Study Combining Case Report, Scoping Review, and Clinical Survey
Source: JIMD Rep. 2025 Sep 25;66(6):e70044. doi: 10.1002/jmd2.70044 (PMC12464341; doi:10.1002/jmd2.70044)
Supplement: Supplementary file 2 — Table S1: Quality assessment for case reports. [file JMD2-66-e70044-s002.docx]

**Supplementary Table 1. Quality assessment for case reports.**

|  | **JBI Critical Appraisal Checklist for Case Reports** | | | | | | | |
| --- | --- | --- | --- | --- | --- | --- | --- | --- |
| **Ref.** | **1** | **2** | **3** | **4** | **5** | **6** | **7** | **8** |
|  | N | N | Y | Y | Y | N | N | Y |
|  | N.A. | N.A. | N.A. | Y | N.A. | N.A. | N.A. | N.A. |
|  | N | Y | Y | Y | N | n | n | Y |
|  | N | N | N | Y | U | N | N | Y |

1. Were patient’s demographic characteristics clearly described?
2. Was the patient’s history clearly described and presented as a timeline?
3. Was the current clinical condition of the patient on presentation clearly described?
4. Were diagnostic tests or assessment methods and the results clearly described?
5. Was the intervention(s) or treatment procedure(s) clearly described?
6. Was the post-intervention clinical condition clearly described?
7. Were adverse events (harms) or unanticipated events identified and described?
8. Does the case report provide takeaway lessons?

Ref: references. Yes: Y. No: N. U: Unclear. Not applicable: N.A.

Moola S, Munn Z, Tufanaru C, Aromataris E, Sears K, Sfetcu R, Currie M, Lisy K, Qureshi R, Mattis P, Mu P. Chapter 7: Systematic reviews of etiology and risk. In: Aromataris E, Munn Z (Editors)*. JBI Manual for Evidence Synthesis.* JBI, 2020. Available from https://synthesismanual.jbi.global. <https://doi.org/10.46658/JBIMES-20-08>

**References**

1. Colović R. Aneurizma slezinske arterije kod bolesnice sa Gaucherovom bolesću [Splenic artery aneurysm in a patient with Gaucher's disease]. *Srp Arh Celok Lek*. 1989;117(1-2):107-113.
2. Agrawal GA, Johnson PT, Fishman EK. Splenic artery aneurysms and pseudoaneurysms: clinical distinctions and CT appearances. *AJR Am J Roentgenol*. 2007;188(4):992-999. doi:10.2214/AJR.06.0794
3. Doğan, E. E., Telci Çaklılı, Ö., Rasulova, N., Karakeçi, S., Hacişahinoğulları, H., Gül, N., ... Soyluk Selçukbiricik, Ö.(2021). Splenic Artery Aneurysm: A Rare Complication of Type 1 Gaucher Disease . Endobridge 2021
4. Klinkert P, Kluit AB, de Vries AC, Puylaert JB. Spontaneous rupture of the spleen: role of ultrasound in diagnosis, treatment, and monitoring. *Eur J Surg*. 1999;165(7):712-713. doi:10.1080/11024159950189807
