# Supplementary material for: Splenic Artery Aneurysm in Gaucher Disease: A Hybrid Study Combining Case Report, Scoping Review, and Clinical Survey
Source: JIMD Rep. 2025 Sep 25;66(6):e70044. doi: 10.1002/jmd2.70044 (PMC12464341; doi:10.1002/jmd2.70044)
Supplement: Supplementary file 3 — Table S2: Quality assessment for case series. [file JMD2-66-e70044-s003.docx]

**Supplementary Table 2. Quality assessment for case series.**

|  | **JBI Critical Appraisal Checklist for Case Series** | | | | | | | | | |
| --- | --- | --- | --- | --- | --- | --- | --- | --- | --- | --- |
| Ref. | 1 | 2 | 3 | 4 | 5 | 6 | 7 | 8 | 9 | 10 |
|  | Y | Y | Y | Y | Y | Y | Y | Y | Y | Y |
|  | Y | Y | N.A. | N | N | Y | Y | Y | N | N.A. |

1. Were there clear criteria for inclusion in the case series?
2. Was the condition measured in a standard, reliable way for all participants included in the case series?
3. Were valid methods used for identification of the condition for all participants included in the case series?
4. Did the case series have consecutive inclusion of participants?
5. Did the case series have complete inclusion of participants?
6. Was there clear reporting of the demographics of the participants in the study?
7. Was there clear reporting of clinical information of the participants?
8. Were the outcomes or follow-up results of cases clearly reported?
9. Was there clear reporting of the presenting sites’/clinics’ demographic information?
10. Was statistical analysis appropriate?

Yes: Y. No: N. Unclear: U. Not Applicable: N.A.

Moola S, Munn Z, Tufanaru C, Aromataris E, Sears K, Sfetcu R, Currie M, Lisy K, Qureshi R, Mattis P, Mu P. Chapter 7: Systematic reviews of etiology and risk. In: Aromataris E, Munn Z (Editors)*. JBI Manual for Evidence Synthesis.* JBI, 2020. Available from https://synthesismanual.jbi.global. https://doi.org/10.46658/JBIMES-20-08

**References**

1. Serratrice C, Cox TM, Leguy-Seguin V, et al. Splenic Artery Aneurysms, A Rare Complication of Type 1 Gaucher Disease: Report of Five Cases. *J Clin Med*. 2019;8(2):219. Published 2019 Feb 8. doi:10.3390/jcm8020219
2. Sherlock S.P.V., Learmonth J.R. Aneurysm of the splenic artery with an account of an example complicating Gaucher’s disease. Br. J. Surg. 1942:151–160. doi: 10.1002/bjs.18003011809.
