# Supplementary material for: Splenic Artery Aneurysm in Gaucher Disease: A Hybrid Study Combining Case Report, Scoping Review, and Clinical Survey
Source: JIMD Rep. 2025 Sep 25;66(6):e70044. doi: 10.1002/jmd2.70044 (PMC12464341; doi:10.1002/jmd2.70044)
Supplement: Supplementary file 4 — Table S3: Characteristics of subjects included in the Scoping review with IPD analysis. [file JMD2-66-e70044-s001.docx]

| **Patients** | **Sex** | **Age at SAA** | **Age at GD diagnosis** | **Genotype** | **Splenomegaly** | **Thrombocytopenia** | **Prior ERT** | **ERT after SAA diagnosis** | **SAA treatment** | **Exitus** |
| --- | --- | --- | --- | --- | --- | --- | --- | --- | --- | --- |
| **1** | F | 63 | 30 | N370S/N370s | YES | YES | NO | - | - | D |
| **2** | F | 49 | 19 | N370S/84GG | YES | YES | YES | - | Splenectomy | A |
| **3** | M | 40 | 10 | N370S/N370S | YES | YES | NO | YES | - | A |
| **4** | M | 58 | 38 | N370S/D218A | YES | YES | NO | - | Embolization | A |
| **5** | M | 29 | 29 | N370S/K196F | YES | YES | NO | YES | - | A |
| **6** | F | 43 | 43 | - | YES | YES | NO | - | Splenectomy | A |
| **7** | F | 31 | - | - | YES | - | NO | - | Splenectomy | A |
| **8** | F | 50 | - | - | - | - |  | - | - | - |
| **9** | M | 56 | 52 | R398L | YES | YES |  | - | - | - |
| **10** | F | 48 | 48 | - | -- | - | NO | - | Splenectomy | A |

**Supplementary Table 3. Characteristics of subjects included in the Scoping review with IPD analysis**

SAA: splenic artery aneurism; GD: Gaucher disease; ERT: enzyme replacement therapy; F: female; M: male; D: died; A: Alive
